# Examining the Roles of Genomic Context and Endogenous Regulatory Elements on IS*1* Transposition Within the *Escherichia coli* Genome

**DOI:** 10.3390/ijms26178375

**Published:** 2025-08-28

**Authors:** Sofia Smith, Zhongge Zhang, Allyson Ho, Tusha Karnani, Jack Ord, Milton H. Saier

**Affiliations:** Department of Molecular Biology, School of Biological Sciences, University of California, 9500 Gilman Dr, La Jolla, San Diego, CA 92093-0116, USA; ssmith@ucsd.edu (S.S.);

**Keywords:** insertion sequence, transposable element, IS*1*, intrinsic sequence, InsA, ribosomal frameshift, genomic context, replicative vs. non-replicative mechanism

## Abstract

Insertion sequence (IS) elements are key drivers of bacterial genome plasticity, yet the overall regulation of their transposition remains poorly understood. This is especially true for the multiple-layer regulation at the donor site, which has been largely overlooked. Using multiple mutation assays, genetic manipulations and reporter genes, this study focuses on characterizing how endogenous DNA sequences, transcriptional and translational factors, and genomic context regulate IS*1* transposition from its donor site. Out of six elements within the chromosome of *E. coli* strain BW25113, IS*1*A and IS*1*E (both with the consensus sequence) contribute to over 99.9% of the overall IS*1* transposition within the genome while the other four elements without the non-consensus sequence are essentially incapable of transposing. Inducing a ribosomal -1 frameshift at the A_6_C motif increases transposition over 1000-fold, but this enhancement is largely reversed by restoring InsA-mediated transcriptional regulation. Strikingly, genomic sequences flanking IS*1* elements appreciably modulate transposition by promoting transcription or facilitating formation of transpososomes, a phenomenon that remains under-studied. Finally, IS*1* was confirmed to undergo replicative transposition intramolecularly, a mechanism shown here to be independent of transposase levels in the cell. These findings contribute to our understanding of mobile genetic element regulation and potentially offer strategies for mitigating their potentially harmful effects.

## 1. Introduction

DNA mobility is a central theme in genome evolution, and transposons (mobile elements) represent one of the most versatile mechanisms in shaping prokaryotic genomes. As the smallest of the transposons, insertion sequence (IS) elements, are capable of intramolecular and intermolecular transposition [1]. They transpose with a protein that they encode themselves, a transposase, and integrate into their target site to create an insertional mutation. This can happen in a replicative or non-replicative manner (conservative): either the IS element is copied such that the original element remains at its original (donor) location while the copy inserts itself into the target (recipient) location (replicative) or the element is removed from its donor location and moved to the recipient location (non-replicative). Transposition of IS elements can yield multiple genomic effects, including chromosomal DNA rearrangements [2,3], deletions [4,5] and gene/operon (in)activation [6,7]. Furthermore, there has been a strong and concerning link between bacterial resistance to antibiotics and IS element movement [8,9].

Thought to have originated in the AT-rich RD-2 head processing region [10] as a constituent of the P1-phage genome [11], IS*1* is a well-studied IS element commonly distributed in Gram-negative enteric bacteria, such as *E. coli* and *Shigella* strains as well as *Salmonella enterica* serovar Typhimurium [12]. Like other IS elements, IS*1* insertion usually leads to major genetic changes, involving gene activation [13,14] or inactivation [7,15], as well as multi-drug resistance in bacterial cells [7,13,14].

**Figure 1:**
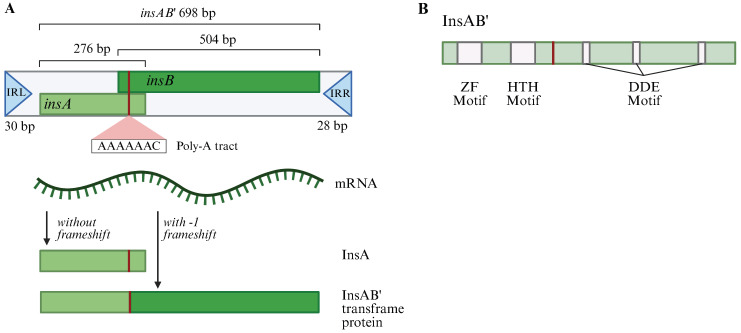
Schematic diagrams for an IS*1* element and its encoded transposase. (**A**) Diagrams showing structural organization of IS*1* and its transcriptional and translational products. IS*1* is 768 bp long and is flanked by two inverted repeat sequences (IRL and IRR). It carries two overlapping open reading frames (*insA* and *insB*) and a transframe gene *insAB*’. The IS*1* transcript can yield two proteins: InsA (with no -1 ribosomal frameshift at the poly-A tract) and InsAB’ (with the -1 frameshift). (**B**) Diagram showing organization of the IS*1* transposase InsAB’. InsAB’ is 232-AA long, carries a ZF motif and an HTH motif at the N-terminus, and a DDE motif at the C-terminus. The first two of these motifs are required for InsA and InsAB’ binding to IRL and IRL while the last one is critical for catalytic activity of InsAB’. Created in BioRender Canvas. Smith, S. (2025) https://BioRender.com/0q4p31r. (accessed on 30 July 2025).

As shown in Figure 1 and Figure S1, IS*1* is the smallest (total of 768 base pairs) among IS elements, with imperfect inverted repeats (IRs) about 30 bp in length at both ends (IRL and IRR denoting the IR at the left side and at the right side, respectively). It carries two overlapping reading frames, *insA* and *insB*, which are transcribed in the same direction and are driven by an extremely weak promoter (P_IS1_) [16,17] that partially overlaps IRL. Near the end of *insA* lies a poly-adenosine (poly-A) tract (AAAAAAC, referred to as the A_6_C motif), which occasionally causes the ribosome to undergo a -1-frameshift before continuing translation (the primary translational regulator of IS*1)*. While *insA* codes for the P_IS1_ repressor InsA (the primary transcriptional regulator of IS*1*), *insB* does not code for a separate protein; rather, the -1 translational frameshift must occur within the A_6_C motif to avoid the *insA* stop codon and allow translation of *insB* to create the transposase InsAB’ necessary for IS*1* transposition (Figure 1A). The frameshift rate due to the A_6_C motif alone is effectively very low; however, the presence of an RNA secondary structure (a “pseudoknot”) downstream of the A_6_C motif in IS*1* [18] may appreciably increase the frameshift rate [19], which also contributes to translational regulation. InsA contains a zinc-finger (ZF) motif and a helix-turn-helix (HTH) motif that are required for its IR-specific DNA binding [16,20,21]. InsAB’ (the transposase) harbors a catalytic DDE motif essential for IS*1* transposition [22] in addition to the ZF and HTH motifs it shares with InsA (Figure 1B). This arrangement allows IS*1* to be compact as InsA and InsAB’ share DNA binding domains. Thus, the relative concentrations of the two proteins are likely critical in determining the transposition frequency. We here dissect the relative roles of these regulations in IS*1* transposition by examining these regulators individually. Lastly, each transposition causes a 8 bp or 9 bp duplication of the target site on either side of the newly inserted IS*1* element [23].

Nucleotide sequences, copy numbers and locations of IS*1* elements are variable amongst different *E. coli* strains [24,25]. In the genome of *E. coli* K12 BW25113 [26] used in this study, there are six IS*1* elements (IS*1*A-IS*1*F) of the same size (768 bp), which are classified into four types based on their unique sequences [27,28]. As a major type of IS*1*, IS*1*A and IS*1*E have the same nucleotide sequence, which is referred to as IS*1*’s “consensus sequence” in this study since these two elements have been used the most in investigation of IS*1* transposition. IS*1*A is located at 20,508 bp just downstream of the *nhaAR* operon (transcribed convergently with respect to IS*1*A). Directly downstream of IS*1*A lies *rpsT* (transcribed in tandem with IS*1*A), followed by a Rho-independent terminator. IS*1*E is located at 3,583,483 bp within the uncharacterized *yrhA* gene (transcribed in tandem with IS*1*E). As a 2nd type, IS*1*B and IS*1*C have the same sequence as well. IS*1*B is within the *afuB* gene, and IS*1*C is located between *ykgS* and *yagJ*. As a 3rd type, IS*1*D is located within the promoter region of *gfcA*. Lastly, as the 4th type, IS*1*F is located just downstream of the *fecABCDE* and *fecIR* operons, transcribed divergently with respect to IS*1*F. While the overall IS*1* transposition process has been somewhat characterized, most investigations have focused on intermolecular transposition (e.g., plasmid to plasmid transposition) [29,30,31]. Thus far, little information is available concerning the activities of IS*1* transposition within a bacterial genome. Essentially no efforts have been devoted to examining the transposition rates of these six IS*1* elements present on the *E. coli* chromosome [32]. Given the slight sequence variability between elements, understanding their individual activities could provide insight into how sequence differences affect transposition efficiency.

For each IS*5* transposition event, two key genomic regions are directly involved: one serving as the recipient site (to receive the incoming IS*5* element) and the other serving as the donor site (to donate the transposing IS*5* element) [33]. Conceivably, the same case applies to IS*1*. Most previous efforts have concentrated on investigating how IS*1* elements insert into their recipient/target sites, ignoring the regulatory layers surrounding the donor site. Consequently, little information is available concerning how the surrounding genomic context and endogenous regulatory factors of IS*1* concurrently impact transposition frequencies within the chromosome. Furthermore, while overall IS*1* transposition frequencies have been characterized, the transposition rates of individual IS*1* elements remain uncharacterized. This is notable given that the sequences of these copies vary slightly, which could influence their transposition activity.

IS*1* elements encode a DDE-type transposase that is characterized by the presence of a conserved Asp-Asp-Glu (DDE) motif serving as its catalytic site [27]. Initiating non-replicative transposition requires an endonuclease to make double stranded breaks [28], while replicative transposition usually only requires single stranded nicking [34]. DDE transposases are known to possess intrinsic nicking-ability. The IS*1* transposase has been speculated to transpose non-replicatively [35] but has been observed to transpose primarily replicatively [30]. However, it is unclear what the primary mechanism could be by which IS*1* transposes (intramolecularly) within a bacterial genome.

This study aims to fill in these gaps, focusing on the IS*1* element. Here, we show that the IS*1* elements maintaining the consensus sequence, IS*1*A and IS*1*E, are the only two out of six elements that significantly contribute to IS*1* transposition. Furthermore, in their native state, IS*1*E transposes at a slightly higher frequency than IS*1*A. Constitutive induction of the -1 ribosomal frameshift at IS*1* drastically increases the transposition frequency for these elements. Overproduction of the repressor InsA nearly abolishes transposition of both native and frameshift-induced IS*1* elements. Genomic contexts flanking IS*1* elements appreciably affect their transposition by exerting stimulatory effects on transcription of the transposase gene or aiding formation of the transpososome. Lastly, we confirm that the primary mechanism of transposition is replicative.

## 2. Results

### 2.1. IS1A and IS1E Maintain the IS1 Consensus Sequence and Are the Primary Contributors to Transposition

**Figure 2:**
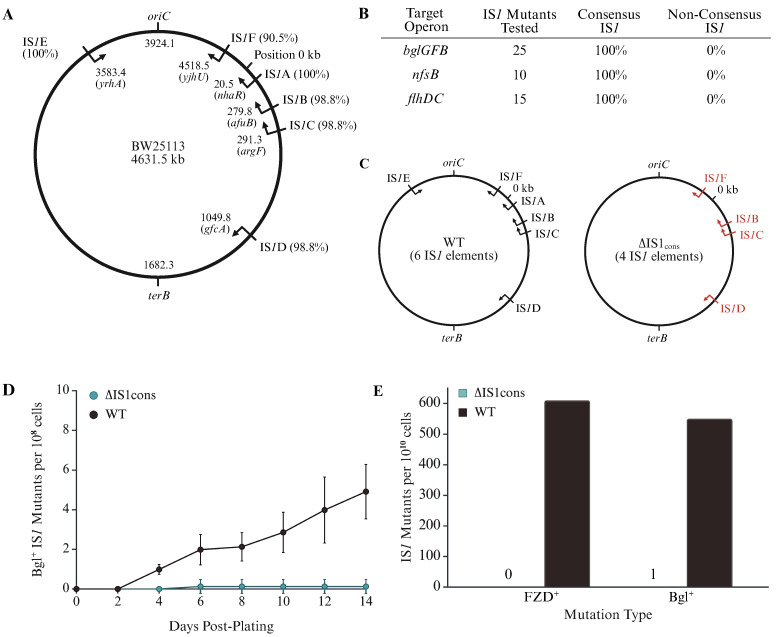
Consensus-sequence IS*1*E and IS*1*A are the primary contributors to overall IS*1* transposition within the genome. (**A**) Schematic diagram showing six IS*1* elements on the chromosome of strain BW25113. Their locations (relative to position 0, and *oriC* and *terB*), orientation (relative to the nearby target genes), and percent identities to the consensus sequence are indicated. (**B**) All analyzed IS*1*-mediated Bgl^+^, FZD^+^ and SWM^+^ mutations were derived from IS*1*E and IS*1*A. (**C**) Diagrams showing all six IS1 elements in WT (**left**) and four IS1 elements in ∆IS1_cons_ (**right**). Strain ∆IS1_cons_ is the same as WT except that IS1E and IS1A are deleted. (**D**) Effect of deleting IS1A and IS1E on IS1-mediated Bgl^+^ mutations over time in a 14-day period (n = 8 and 7). (**E**) IS1 transposition is nearly abolished in strain ∆IS1_cons_. Both Bgl^+^ and FZD^+^ mutation assays were conducted to determine if four non-consensus sequence IS1 elements (IS1B, IS1C, IS1D and IS1F) were capable of transposition into the bglGFB and nfsB targets in a 14-day period. Created in BioRender Canvas. Smith, S. (2025) https://BioRender.com/krg9vh5. (accessed on 21 August 2025).

There are six IS*1* elements (IS*1*A, IS*1*B, IS*1*C, IS*1*D, IS*1*E and IS*1*F) representing four unique types [28] on the chromosome of *E*. *coli* K12 strain BW25113 [26]. Type I includes IS*1*A and IS*1*E, and their nucleotide sequences are identical (Figure S1). For each of these IS*1* elements, its chromosomal location, neighbor gene, orientation (relative to the nearby genes) and similarity to the consensus sequence are shown on Figure 2A. In this study, we define this 768 bp sequence as the IS*1* consensus sequence (cons) since these two elements are the dominant contributors for overall IS*1* transposition. Type II contains IS*1*B and IS*1*C with identical sequences, which reveal 9 mismatches relative to the consensus sequence (Figure S2). IS*1*D (type III) harbors 9 mismatches, 7 of which are the same as in type II, while IS*1*F (type IV) harbors 73 mismatches as compared to the consensus sequence (Figure S1). The sequences of all six IS*1* elements were confirmed by DNA sequencing. As shown in Figure S2, most of the mismatches were found within the coding region of *insA* or *insAB’*.

To identify whether non-consensus sequence (non-cons) elements can transpose, we first determined which types of IS*1* element were capable of transposition within the genome using strain WT. Twenty-five independent IS*1*-mediated Bgl^+^ mutants, isolated from β-glucoside growth (Bgl^+^) mutation assays subject to sequencing analyses, carried the IS*1* consensus sequence (i.e., IS*1*A or IS*1*E from type I) present upstream of the *bglGFB* promoter (P*_bgl_*) region. Similar sequence analyses were conducted using ten IS*1* insertional FZD^+^ mutants and fifteen SWM^+^ mutants, and all these mutants carried type I IS*1* elements at their respective recipient site (Figure 2B). These results strongly suggest that IS*1*B/IS*1*C (type II), IS*1*D (type III) and IS*1*F (type IV), each having a non-consensus sequence, are either unable to transpose or transpose at an extremely low frequency.

To further examine the transposition activity of non-cons IS*1* elements, strain ∆IS1_cons_ was made, in which the two type I elements (IS*1*A and IS*1*E) were deleted leaving only four non-consensus elements (IS*1*B, IS*1*C, IS*1*D and IS*1*F) (right diagram of Figure 2C). Using Bgl^+^ mutation assays and FZD^+^ mutation assays, this strain was compared with the WT strain (left diagram of Figure 2C) for IS*1* transposition upstream of P*_bgl_* and within *nfsB*, respectively. IS*1* insertional Bgl^+^ mutations arose from WT plates beginning on day 2 post inoculation, and continued arising with time. However, no IS*1*-mediated Bgl^+^ mutations were detected from the plates with strain ∆IS1_cons_ (IS*1*A/IS*1*E deleted) during a 2-week incubation (Figure 2D). By using numerous plates and multiple Bgl^+^ assays, only one singular instance of an IS*1* transposition event from ∆IS1_cons_ cells (Figure 2E) was observed, making the rate of transposition for non-cons copies 1 per 10^10^ cells (over the course of two weeks), and was thus nearly non-detectable. Meanwhile, no IS*1* insertional FZD^+^ mutations were detected on the *nfsB* target from ∆IS1_cons_ cells although the same mutation readily occurred with WT cells (Figure 2E).

Based on the above results, we conclude: (i) the two type I IS*1* elements with consensus sequence, IS*1*A and IS*1*E, make up nearly all the transpositions responsible for IS*1* insertions seen in our assays, while the other four non-cons IS*1* elements (IS*1*B, IS*1*C, IS*1*D and IS*1*F) are capable of transposing, but with extremely low frequencies; (ii) the endogenous nucleotide sequences embedded within IS*1* significantly affect its transposition activity, and (iii) the mismatches in IS*1*B, IS*1*C, IS*1*D and IS*1*F seem to abolish or drastically minimize production of a functional transposase.

### 2.2. IS1E Transposes at a Higher Frequency than IS1A Although Both Are Main Contributors for IS1 Transposition

**Figure 3:**
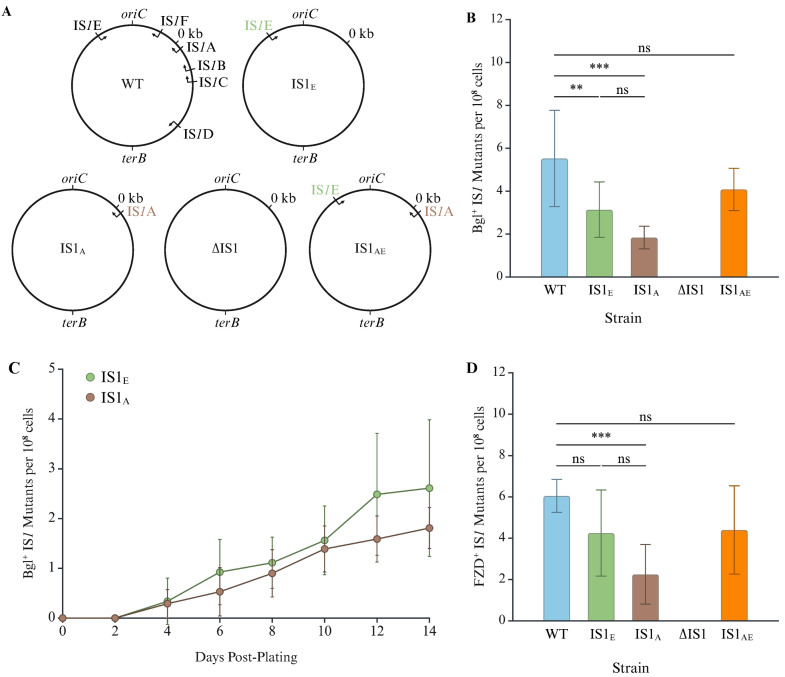
IS1E transposes more frequently than IS1A. (**A**) Diagrams displaying the IS1 elements present in five strains. WT carries all six IS1 elements, with IS1_E_ only retaining IS1E, IS1_A_ only retaining IS1A, ∆IS1 deleted for all six IS1 elements, and IS1_AE_ only retaining IS1A and IS1E. (**B**) Bgl^+^ mutation assays for IS1 insertion comparing IS1_E_, IS1_A_ and IS1_AE_ with WT (n = 8 or 10). (**C**) Bgl^+^ mutations over time comparing IS1_E_ and IS1_A_ (n = 8). (**D**) FZD^+^ mutation assays for IS1 insertion comparing IS1_E_, IS1_A_ and IS1_AE_ with WT (n = 8). Data are plotted as the mean ± SD (one-way ANOVA with Tukey multiple comparisons test or (**B**) one-way ANOVA with Games-Howell multiple comparisons test (**D**)). ns denotes no significance and indicates a *p*-value ≥ 0.05; ** indicates a *p*-value < 0.01; *** indicates a *p*-value < 0.001. Created in BioRender Canvas. Smith, S. (2025) https://BioRender.com/5qgrkh0. (accessed on 21 August 2025).

Having shown that IS*1*A and IS*1*E, both with the consensus sequence, are the primary sources of IS*1* transposition in K12 BW25113, we moved on to determine whether one of them was the main contributor to transposition. To do this, several IS*1* deletion strains were made: IS1_E_ (only retaining IS*1*E at the *yrhA* locus), IS1_A_ (only retaining IS*1*A at the *nhaR* locus), IS1_AE_ (only retaining IS*1*E and IS*1*A), and ∆IS1 (having all six copies of IS*1* deleted) (Figure 3A).

The frequencies of IS*1* transposition into the regulatory region of the *bglGFB* operon were measured for these strains using the Bgl^+^ assay (Figure 3B). As expected, the ∆IS1 strain did not result in IS*1* transposition, confirming successful removal of all IS*1* elements from the chromosome. There was no statistical difference in the frequency of IS*1* transposition between the wild-type (WT) strain and the IS1_AE_ strain, confirming that the two consensus copies of IS*1* make up most, if not all, of the IS*1* transpositions in WT cells. Both single copy IS*1* strains, IS1_E_ and IS1_A_, exhibited a decreased frequency of IS*1* transposition (by about half compared to the WT) although somewhat more frequent IS*1* insertions were observed for strain IS1_E_ than for strain IS1_A_. To further examine the transposition activity of IS1_E_ and IS1_A_, we ran a separate Bgl^+^ mutation assay over time (Figure 3C). Once again, IS1_E_ yielded slightly more IS*1* insertional Bgl^+^ mutants than IS1_A_ although the differences may not be significant on some days (as demonstrated by the overlapping standard deviations).

The same strains were tested for IS*1* transposition using the FZD^+^ assays to validate these trends at another locus: *nfsB*. Similar observations were made: WT and IS1_AE_ had similar frequencies of transposition while IS1_E_ had more FZD^+^ mutants than IS1_A_ (Figure 3D). These findings suggest that neither copy (IS*1*A nor IS*1*E) alone is exclusively responsible for overall IS*1* transposition, but IS*1*E transposes slightly more frequently than IS*1*A.

### 2.3. Translational Regulation Has a Large Effect on IS1 Transposition

**Figure 4:**
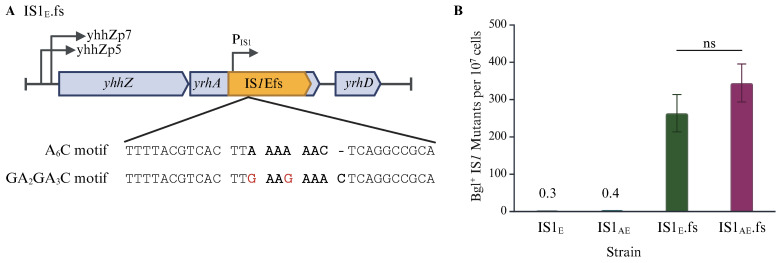
Ribosomal frameshift plays a critical role in IS*1* transposition. (**A**) Schematic diagram depicting the A_6_C motif and the altered GA_2_GA_3_C motif at IS*1*E. The sequences for both motifs are bolded. 1 bp (G) insertion and 1 bp (G) substitution, which eliminate the -1 frameshift requirement for the synthesis of InsAB’, are in red at the GA_2_GA_3_C motif. Three codons near the slippery sequence are separated by spaces in each motif. (**B**) Effect of eliminating translational regulation by modifying the A_6_C motif on IS*1* transposition in IS1E.fs (n = 6) and IS1AE.fs (n = 4). Data are plotted as the mean ± SD (Welch’s one-way ANOVA with Dunnett T3 multiple comparison’s test. ns denotes no significance and indicates a *p*-value ≥ 0.05. Created in BioRender Canvas. Smith, S. (2025) https://BioRender.com/a34lvbk. (accessed on 30 July 2025).

IS*1* transposase production is subject to both transcriptional control (mediated by the repressor InsA) and translational control (mediated by ribosomal frameshift at the A_6_C motif). To characterize the translational regulation, we removed the translational requirement in strain IS1_E_ by changing the A_6_C motif (AAAAAAC) to the GA_2_GA_3_C motif (GAAGAAAC), yielding strain IS1_E_.fs (Figure 4A). Such alterations caused the default expression of the transposase gene *InsAB*’ while abolishing repressor gene *insA* expression. The same changes were made on the IS*1*E element in strain IS1_AE_ (retaining IS1A and IS1E) to create strain IS1_AE_.fs, which carries the native IS*1*A (with the original A_6_C motif) and the induced frameshift at IS*1*E (with the GA_2_GA_3_C motif). Since IS*1* transposition is dependent on transcriptional and translational regulation, these new strains would allow us to separate the two and measure the extent of the translational regulation by removing it.

Using Bgl^+^ mutation assays, these frameshift-induced strains were compared with the relative native strains for IS*1* transposition into the *bglGFB* target (Figure 4B). The IS*1* transposition frequencies in IS1_E_.fs (with a constitutively induced frameshift in the A_6_C motif) increased from about 0.3 mutants to 270 mutants per 10^7^ cells, a 900-fold elevation. Strain IS1_AE_.fs, carrying the frameshift-induced IS*1*E element, IS*1*Efs, together with the native IS*1*A, exhibited a similar increase in transposition over strain IS1_AE_ (0.4 mutants for IS1_AE_ vs. 330 mutants for IS1_AE_.fs). It is worth noting that strain IS1_AE_.fs had a somewhat greater insertion frequency than IS1_E_.fs (330 mutants for IS1_AE_.fs vs. 270 mutants for IS1_E_.fs). This could be due to the *trans* effect of the transposases over-produced by IS*1*Efs on IS*1*A transposition (see Section 3). This drastic increase, due to the removal of translational regulation, indicates that translational regulation plays an important and large role in repressing IS*1* transposition.

### 2.4. Transcriptional Regulation Has a Large Effect on IS1 Transposition

**Figure 5:**
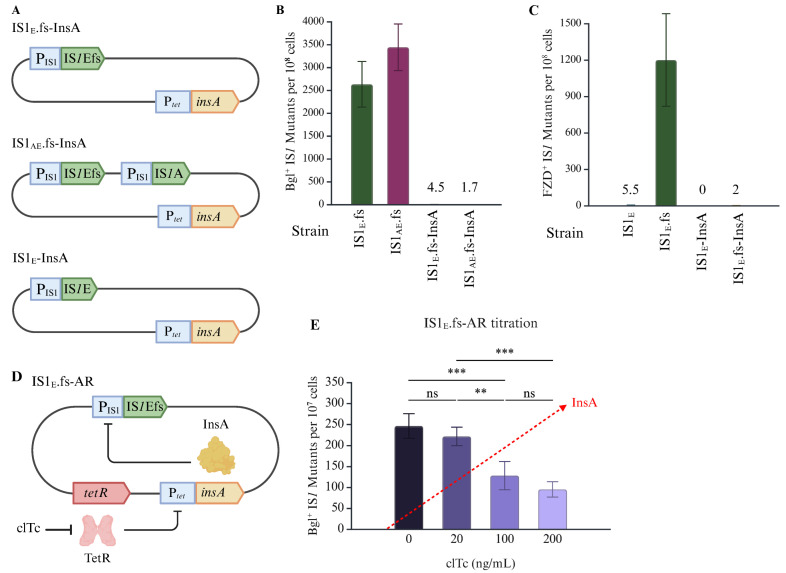
InsA overexpression significantly diminishes IS*1* transposition. (**A**) Diagram showing the P*_tet_* driven *insA* cassette at the *intS* locus in strains IS1_E_.fs-InsA, IS1_AE_.fs-InsA, and IS1_E_-InsA. InsA is over produced in these strains. (**B**) Effect of *insA* overexpression on IS*1* transposition into the *bglGFB* (from left to right, n = 6, 4, 5, and 5). (**C**) Effect of *insA* overexpression on IS*1* transposition into *nfsB* (from left to right, n = 8, 10, 6, and 6). (**D**) Diagram showing the titratable regulatory cascade introduced on the chromosome of IS1_E_.fs. In this cascade, TetR represses P*_tet_* driven *insA* and the repression can be relieved by adding chlortetracycline (clTc), with more clTc leading to greater levels of InsA. (**E**) The frequencies of IS*1* insertion into the *bglGFB* target in IS1_E_.fs-AR is inversely proportional to InsA levels (n = 4, 4, 4, and 5). Data are plotted as the mean ± SD (One-way ANOVA with Tukey multiple comparison’s test (**E**)). ns denotes no significance and indicates a *p*-value ≥ 0.05; ** indicates a *p*-value < 0.01; *** indicates a *p*-value < 0.001. Created in BioRender Canvas. Smith, S. (2025) https://BioRender.com/t2yd08s.(accessed on 30 July 2025).

Due to the alterations at the A_6_C motif, frameshift-induced IS*1* elements lose the ability to express the repressor gene *insA*. To examine the transcriptional regulation affecting IS*1* transposition, an expression cassette that constitutively expressed *insA* at a separate *intS* locus using a strong promoter (P*_tet_*) was introduced back to IS1_E_, IS1_E_.fs, and IS1_AE_.fs to create IS1_E_-InsA, IS1_E_.fs-InsA, and IS1_AE_.fs-InsA (Figure 5A). This set-up (with the transcriptional repression and the translational frameshift control at two distinct loci) allowed us to investigate the transcriptional effect alone on IS*1* transposition using both native IS*1* strains and frameshift-induced strains.

The frequencies of IS*1* transposition into the *bglGFB* and *nfsB* target sites were quantitated for these strains using Bgl^+^ and FZD^+^ assays. The addition of InsA to IS1_E_.fs and IS1_AE_.fs reduced IS*1* transposition into the *bglGFB* operon by about 1000x, down to native state transposition levels (Figure 5B). Similar effects were observed for transposition into the *nfsB* gene. As shown in Figure 5C, the *insA* overexpression abolished IS*1*E transposition (5.5 FZD^+^ mutants to 0 mutants per 10^8^ cells) (see strain IS1_E_ in the 1st and 3rd columns), emphasizing how sensitive IS*1* transposition is to InsA. This overexpression led to an ~600x decreased transposition frequency for strain IS1_E_.fs (2nd and 4th columns on Figure 5C). These results highlight the potency of InsA as a repressor for the IS*1* promoter P_IS1_ and the strength of transcriptional regulation in controlling IS*1* transposition activity. Of note, InsA not only represses transcription, but also directly represses IS*1* transposition by competing with InsAB’ binding to both IRL and IRR.

To vary production of InsA, thus proportionally controlling IS*1* transposition activity, a regulatory cascade was created by constitutively expressing *tetR*, the repressor for P*_tet_*, in strain IS1_E_.fs-InsA. The resultant strain IS1_E_.fs-AR harbors three modules, IS1Efs (frameshift-induced IS1E), P*_tet_* driven *insA*, and constitutively expressed *tetR* (Figure 5D). This cascade is titratable with the small molecule chlortetracycline (clTc), such that with increasing amounts of clTc, more InsA is produced, and less transposition is expected (Figure 5D). The intended effect was observed; increasing levels of clTc led to a reduction in transposition, supporting the proposed model (Figure 5E). Of note, the addition of clTc at 200 ng/mL only reduced IS*1* transposition 2.5-fold as compared to the treatment without clTc, indicating that the *insA* gene was insufficiently expressed at this level of clTc due to the strong repression by TetR. Though these experimental conditions use much higher levels of InsA and InsAB’ than the native state does, the titratability is adequately demonstrated and shows that IS*1* transposition activity is inversely correlated with the levels of InsA.

As described above, the availability of transposases, essential for IS*1* transposition, is highly dependent on both transcriptional regulation (mediated by InsA) and translational regulation (mediated by the -1 ribosomal frameshift), implying that IS*1* transposition is extremely tightly controlled. Given the potential for genomic disruption, tight regulation makes sense due to the fact that frequent IS*1* transposition is likely harmful to the cell: a notion further supported by evidence from other studies [36,37].

### 2.5. Effects of Genomic Contexts on IS1 Transposition

**Figure 6:**
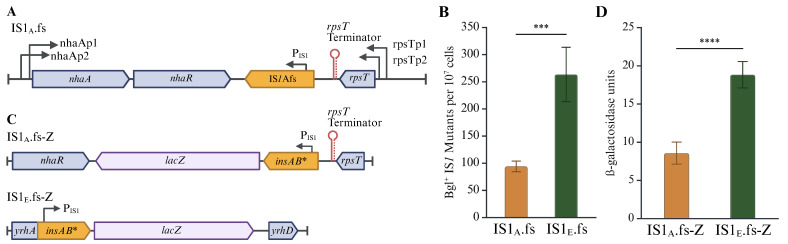
IS*1*Efs not only transposes more frequently but also synthesizes more IS*1* transcripts than IS*1*Afs. (**A**) Schematic diagram showing genomic contexts surrounding IS1_A_.fs. (**B**) Bgl^+^ mutation assays comparing IS*1*Afs and IS*1*Efs for their transposition activities (n = 9 and 6, respectively). (**C**) Schematic diagrams showing the *lacZ* transcriptional reporter in strains IS1_A_.fs-Z and IS1_E_.fs-Z. Both reporters have the same P_IS1_ promoter and the same frameshift-induced transposase gene insAB*, but with different genomic contexts. (**D**) β-Galactosidase assays comparing transcription levels between IS1_A_.fs-Z and IS1_E_.fs-Z. Data are plotted as the mean SD (Welch’s *t*-test (**B**) and unpaired *t*-test (**D**)). *** indicates a *p*-value < 0.001; **** indicates a *p*-value < 0.0001. Created in BioRender Canvas. Smith, S. (2025) https://BioRender.com/r6rx2bp. (accessed on 21 August 2025).

As shown in Section 2.2, two native IS*1* elements with identical sequences, IS*1*E at the *yrhA* locus and IS*1*A at the *nhaR* locus, exhibited moderately different transposition activity with IS*1*E transposing at a higher frequency than IS*1*A (Figure 2), suggesting that the genomic DNA contexts play a role in IS*1* transposition. Since all native IS*1* elements seldom transpose, comparing the IS*1*A and IS*1*E elements in their frameshift-induced state is necessary to observe an influence exerted by the genomic context, since these modified elements transpose at much greater frequencies. To do this, the same alteration to the A_6_C motif was introduced into IS*1*A in strain IS1_A_ to create strain IS1_A_.fs (Figure 6A). Using Bgl^+^ mutation assays, the activities of IS*1* transposition into the *bglGFB* target in IS1_A_.fs and IS1_E_.fs were measured by quantitating the insertion frequencies. Strikingly, the IS*1* transposition frequency in IS1_A_.fs was only half that of IS1_E_.fs (Figure 6B). Given that removal of the translational regulation and that IS*1*A and IS*1*E are sequentially identical, this result indicates that the genomic context, being the only differentiating factor, clearly influences IS*1* transposition.

To measure the transcriptional activity of IS*1*A and IS*1*E, a *lacZ* reporter was added downstream of the transposase gene, insAB* (*insAB*’ with the altered A_6_C motif), in strains IS1_A_.fs and IS1_E_.fs (so that *insAB*’ and *lacZ* form an operon) to create IS1_A_.fs-Z and IS1_E_.fs-Z (Figure 6C). β-galactosidase assays were conducted by culturing these reporter strains in minimal glycerol media. As shown in Figure 6D, a similar trend in transcriptional activity was revealed as seen with transposition frequency: IS1_E_.fs presented twice as much transcriptional activity as IS1_A_.fs. With this result, we conclude that the local genomic regions exert a greater transcriptional effect on IS*1*E than IS*1*A since both elements have the same nucleotide sequence but different surrounding chromosomal regions. This result supports the notion that the different transposition activities observed between IS*1*E and IS*1*A or between IS*1*Efs and IS*1*Afs may be at least partially due to their different *insAB*’ transcription activities.

**Figure 7:**
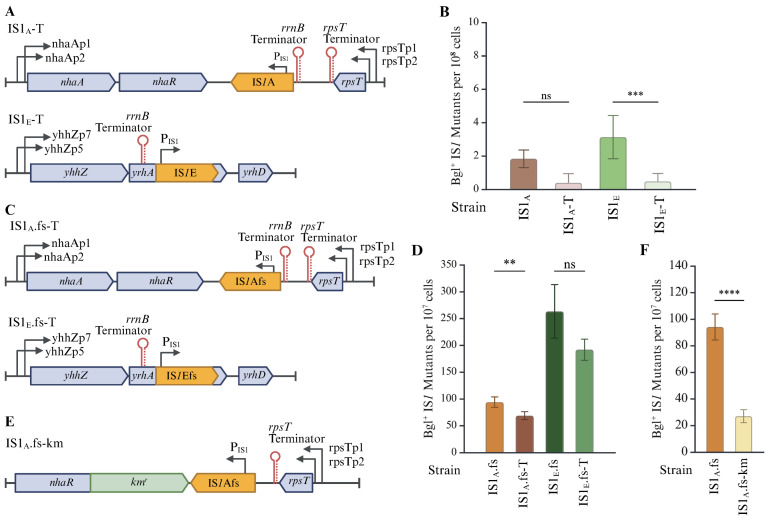
IS*1* transposition is affected by genomic contexts. (**A**) Diagrams showing insertion of an *rrnB* terminator (*rrnB*T) upstream of IS*1*A in strain IS1_A_-T or upstream of IS*1*E in strain IS1_E_-T. (**B**) Upstream *rrnB*T insertion significantly decreases the frequencies of transposition of both native elements (IS*1*A and IS*1*E) into the *bglGFB* target (n = 10, 5, 8 and 7). (**C**) Diagrams showing *rrnB*T insertion upstream of IS*1*Afs in strain IS1_A_.fs-T or upstream of IS*1*Efs in strain IS1_E_.fs-T. (**D**) Upstream *rrnB*T insertion decreases the frequencies of transposition of frameshift-induced elements IS1A.fs and IS1E.fs into the *bglGFB* target (n = 9, 5, 6 and 6). (**E**) Diagram showing insertion of a *km^r^* gene downstream of IS*1*Afs in strain IS1_A_.fs-km. (**F**) Effect of inserting a *km^r^* gene downstream of IS*1*Afs on its transposition into the *bgl* target (n = 9 and 6). Data are plotted as the mean ± SD (Kruskal–Wallis test with Dunn’s multiple comparisons test (**B**), Welch’s one-way ANOVA with Dunnett T3 multiples comparison test (**D**), or Welch’s *t*-test (**F**)). ns denotes no significance and indicates a *p*-value ≥ 0.05; ** indicates a *p*-value < 0.01; *** indicates a *p*-value < 0.001; **** indicates a *p*-value < 0.0001. Created in BioRender Canvas. Smith, S. (2025) https://BioRender.com/3taenvy. (accessed on 21 August 2025).

For a more exact look at whether overflowing transcription from upstream genomic regions influenced IS*1* transposition, a strong terminator, the *rrnB* terminator (*rrnB*T), was added directly upstream of IS*1*A and IS*1*E in two native strains to create IS1_A_-T and IS1_E_-T (Figure 7A). The presence of *rrnB*T (conceivably blocking upstream transcription through the *insAB’* gene) significantly brought down the frequencies of IS*1* insertion into the *bglGFB* operon (Figure 7B), consistent with the previous observation that genomic DNA contexts positively impacted *insAB*’ transcription (Figure 6D).

Since the levels of transposition for IS1_A_-T and IS1_E_-T were so low, further confirmation was sought using the induced-frameshift versions of these two strains. The same *rrnB*T was inserted upstream of IS*1*Afs and IS*1*Efs to create IS1_A_.fs-T and IS1_E_.fs-T (Figure 7C). As can be seen in Figure 7D, IS*1* transposition activity was moderately reduced by about 30% in these two frameshift-induced strains by blocking upstream transcription, confirming that the *rrnB* terminator does have an inhibitory effect. These results are consistent with the previous observation that genomic DNA contexts positively impact *insAB*’ transcription (Figure 6D).

To determine the possible effect of downstream regions on IS*1* transposition, a 1.2 kb *km^r^* tag was inserted downstream of the IS*1*Afs element in strain IS1_A_.fs to create IS1_A_.fs-km (Figure 7E). The *km^r^* gene, oriented in the opposite direction as *insAB*’, provided a sizable interruption to the region. Bgl^+^ mutation assays were performed to evaluate whether such a genomic disruption would affect IS*1* transposition frequency, comparing IS1_A_.fs-km and IS1_A_.fs. The downstream region disruption by insertion of a *km^r^* tag resulted in a 3× reduction in IS1Afs transposition activity (Figure 7F), indicating that the downstream genomic DNA is important for IS*1* transposition.

Summarizing the above results, the following conclusions can be drawn: (i) the same IS*1* element can transpose at different frequencies when situated at different chromosomal loci; (ii) upstream genomic regions can help to promote transcription of IS*1* by transcribing across the downstream transposase gene *insAB’*; (iii) downstream regions are important for IS*1* transposition as well, potentially by assisting formation of transpososomes; and (iv) different genomic contexts seem to affect IS*1* transposition using different mechanisms.

### 2.6. IS1 Mainly Uses a Replicative Mechanism for Transposition Within the Genome

DDE-type transposases are capable of both replicative and non-replicative transposition [38]. The IS*1* transposase (a DDE transposase) has been speculated to transpose non-replicatively [35] but observed to transpose primarily replicatively [30]. Several studies have made these observations based on intermolecular (plasmid to genome or plasmid to plasmid) transposition [30,39] as opposed to intramolecular transposition. Given the lack of definitive answers, the mechanism being employed in this study during intrachromosomal transposition under stress-induced conditions was identified.

**Figure 8:**
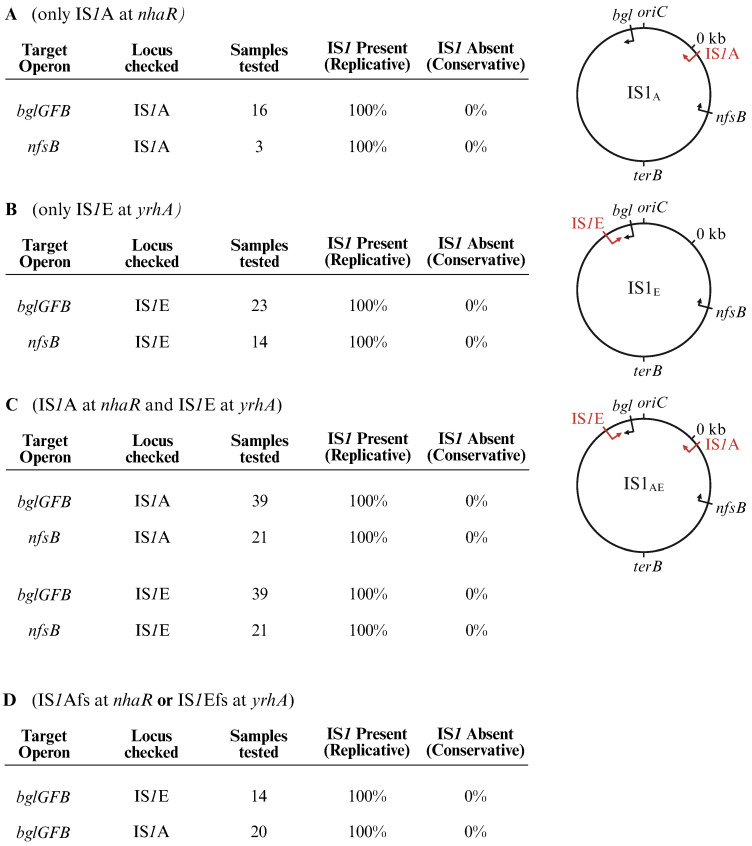
IS*1* transposition mainly uses the replicative mechanism. Independent IS*1* insertional mutants isolated from either Bgl^+^ or FZD^+^ mutation assays for four test strains were used to examine their transposition mechanisms (replicative vs. non-replicative). (**A**) IS*1*A insertional Bgl^+^ and FZD^+^ mutants derived from strain IS1_A_ (retaining IS*1*A only). (**B**) IS*1*E insertional Bgl^+^ and FZD^+^ mutants derived from strain IS1_E_ (retaining IS*1*E only). (**C**) IS*1*A or IS*1*E insertional Bgl^+^ and FZD^+^ mutants derived from strain IS1_AE_ (retaining IS*1*A and IS*1*E). (**D**) IS*1*Afs or IS*1*Efs insertional Bgl^+^ mutants derived from strain IS1_A._fs (retaining IS*1*Afs only) or from strain IS1_E_.fs (retaining IS*1*Efs only). For each of these test IS*1* mutants, the presence of the original IS*1* element(s) at its/their native locus indicates replicative transposition, whereas its/their absence suggests non-replicative transposition. Created in BioRender Canvas. Smith, S. (2025) https://BioRender.com/uux8oxt. (accessed on 30 July 2025).

IS*1* transposed only using the replicative mechanism in this study. To come to this conclusion, independent IS*1* insertional mutants derived from strains IS1_A,_ or IS1_AE_ via Bgl^+^ and FZD^+^ mutation assays were used to examine whether the original element(s) was/were still present in its/their native location(s). If present, the mechanism must have been replicative, and if not, the mechanism must have been non-replicative. 176 new IS*1*-mediated mutants (involving 117 Bgl^+^ mutants and 59 FZD^+^ mutants), which were derived from three distinct parental strains carrying one or two IS*1* elements, were examined and all had the original IS*1* elements situated in their native loci (Figure 8A–C), clearly demonstrating that all these new insertional events resulted from replicative transposition.

To further support the above conclusion, the same procedure was followed for another 34 IS*1* insertional Bgl^+^ mutants from two frameshift-induced strains (IS1_E_.fs and IS1_A_.fs) which transposed at higher frequencies. Again, all samples examined retained the original IS*1* elements at their native loci (Figure 8D). These data provide strong evidence that IS*1* (primarily) employs a replicative mechanism for intra-chromosomal transposition, regardless of the level of transposase present in the cell. Therefore, the replicative mechanism is IS*1*’s dominant mode for all types of transposition, either intermolecular or intramolecular. However, our results cannot rule out the possibility of non-replicative transposition occurring under all conditions.

## 3. Discussion

As a model transposable DNA element, IS*1* transposition and its regulation have been extensively investigated in the past decades. However, most research has focused on the regulatory mechanisms behind IS*1* transposition into its target site (recipient site) during intermolecular transposition [29,30,31,40,41]. While regulation at the target site is important, the multi-level regulation at the donor site (which provides the IS*1* element) also plays a critical role that has been largely overlooked. Thus far, little is known about how the internal nucleotide sequences, both transcriptional and translational controls, and the genomic contexts that flank the IS*1* element, collectively influence transposition activity within a bacterial genome. In this study, we reveal that intrinsic nucleotide sequences are vital for efficient IS*1* transposition from one chromosomal location to another. Transposition is tightly regulated by InsA-mediated transcriptional control and ribosomal frameshift-mediated translational control, both of which repress transposition activity about 1000-fold. Lastly, genomic contexts flanking the element efficiently impact IS*1* transposition either by promoting transcription of the transposase gene or possible by facilitating formation of the transpososomes. To the best of our knowledge, most of these discoveries had not been shown previously on IS*1* intra-genomic transposition. In addition to *E. coli* strains, IS*1* elements are widely present not only in other eubacteria but also in archaea, especially in multiple-antibiotic resistant clinical isolates [42,43]. Similar ribosomal frameshift events are known to occur in the members of the IS*3*, IS*5* and IS*150* families [44,45]. The regulatory mechanism governing IS*1* transposition, elucidated in this study, would likely be applicable to other IS elements, regardless of their origins.

We showed that two out of the six IS*1* elements, IS*1*E and IS*1*A, present in *E. coli* strain BW25113, are capable of regular transposition within the genome. These two elements share the same nucleotide sequence, termed the consensus sequence in the present study. The remaining four elements, IS*1*B, IS*1*C, IS*1*D and IS*1*F, maintaining 90.5% to 98.8% similarity to the consensus sequence (Figure 2A and Figure S2), are incapable of transposing, or transpose at extremely low rates in their native states. There are several possible reasons why these non-consensus sequence IS*1* elements lose their transposing ability. Sequence analyses revealed that several nucleotide substitutions present in IS*1*B, IS*1*C, and IS*1*D, and many in IS*1*F bring about the changes in corresponding amino acid residues, likely giving rise to non-functional or less-functional transposases. For example, the L81F mutation (present in 3 non-cons elements), is situated in the HTH DNA-binding domain, thereby perhaps limiting transposase activity. As introduced earlier, native IS*1* elements synthesize little transposase due to immensely tight control [21] and low promoter activity [17]. Therefore, the low amounts of transposases produced by other elements such as IS*1*E and IS*1*A may be insufficient to transpose the non-consensus sequence elements. Furthermore, and potentially more importantly, it has been reported that IS*1* transposases only function efficiently in *cis* at their native levels [40]*,* being biased to transpose the element that produces the enzyme [46]. Further study into how the non-consensus sequence IS*1* elements respond to larger amounts of transposase would reveal more insight into their transposition. Alternatively, the chromosomal DNA regions surrounding these four elements may somehow prevent their transposition, perhaps by impeding the assembly of transpososomes, as other host regulatory factors influencing transposition, like IHF [17,47,48] and HN-S [49], have been shown to be involved. Lastly, our current experimental conditions may disfavor transposition of these IS*1* elements. Future studies would be needed to investigate the mechanistic details that prevent these four IS*1* elements from transposing. Alternatively, one might look at the appropriate environmental conditions favoring their transposition.

Our work showed that a 1 bp insertion plus 1 bp substitution into the “slippery” A_6_C motif near the 3′ end of *insA* led to an about 1000-fold greater level of transposition. Such a dramatic increase appears to be attributable to the maximal production of the transposase since the rare -1 frameshift is not necessary for InsAB’ synthesis and the InsA repressor is not produced. Several reports revealed that the ratio of InsA to InsAB’, not the absolute levels of IS*1* transcripts, determine the frequencies of transposition [18,50]. For a native IS*1* element to produce InsAB’, ribosomes must exercise a -1-frameshifting event at the A_6_C motif of the IS*1* transcripts [50]. While the slippery A_6_C region is required for the frameshift, a pseudoknot (roadblock) structure formed by a folded downstream segment may also facilitate the frameshift by stalling the ribosome [51]. Therefore, the level of InsAB’ would be exclusively dependent on the frequency of this ribosomal frameshift and influenced by a pseudoknot once the IS*1* transcripts become available. Using a plasmid system expressing *insAB*’, Chandler et al. reported that the -1-frameshifting event occurred in about 2% of the IS*1* transcripts [18], indicative of a rare frequency of the frameshift. Ideally, a more precise approach measuring the frameshift efficiency would be to employ a sensitive translational fusion system on the chromosome, with an out-of-frame reporter gene (such as *lacZ*) fused to the 3′ end of a stop codon-less *insAB*’ gene (with its native promoter or a strong constitutive promoter). In addition to these *cis*-regulatory factors (slippery sequence and roadblock structure), the efficiency of -1 frameshift is modulated by cellular *trans*-acting factors such as proteins and micro-RNAs [52] as well as non-cellular factors such as antibiotics [53] in prokaryotic organisms. Future studies will be directed towards identifying both cellular and non-cellular factors that affect the efficacy of frameshifting and explore how these factors impact ribosomal frameshifts on the A_6_C motif of IS*1*.

Expression of *insA* driven by P*_tet_* at a separate chromosomal locus (*intS*) nearly abolished the intragenomic transposition of not only native-state IS*1* elements but also the frameshift-induced elements (therefore maximally expressing *insAB’*). These results indicate that InsA, sharing the DNA-binding domain with InsAB’, is an incredibly efficient, competitive repressor, not only minimizing IS*1* transcription but also outcompeting InsAB’ for binding to both IRL and IRR of IS*1*. Meanwhile, it is worth noting that P*_tet_* is a stronger promoter than the native IS*1* promoter P_IS1_, which mediated synthesis of a greater than normal amount of InsA, thus strengthening its inhibitory capability.

In the case of any native IS*1*, P_IS1_ and InsA comprise an autorepression system since P_IS1_ drives *insA* (and *insAB’*) transcription, and meanwhile InsA represses P_IS1_. The actual titration for the *insA*/*insAB’* transcription is not clear since the inducer that releases InsA from P_IS1_ is unknown. To titrate *insA* expression (thus varying IS*1* transposition), we used the established P*_tet_*/TetR system, in which *insA* is driven by P*_tet_* while TetR (which was constitutively expressed) represses P*_tet_*, and its binding to P*_tet_* is released by adding the inducer clTc. Using this system, IS*1* transposition frequencies were successfully titrated, with the larger amounts of InsA leading to less frequent IS*1* transpositions. On the other hand, the addition of a large amount (200 ng/mL) clTc only reduced the frequency of IS*1* insertion by 2.5-fold (Figure 5E). This was expected because not enough InsA was produced due to the strong repression of P*_tet_* (driving *insA*) by TetR. A greater transposition reduction could have resulted by adding more clTc, but any levels higher than 200 ng/mL would be toxic to the cells. To improve the P*_tet_*/TetR system, a weaker promoter could be used to drive *tetR*, which would yield higher levels of InsA, thus, more efficiently repressing IS*1* transposition.

Recently, upstream genomic contexts have been shown to exert appreciable effects on transposition of IS*5* by promoting its transposase gene transcription [33,54], another DDE-type IS element that does not have a self-repressor, nor its own complete promoter [55]. A similar stimulatory effect was observed for IS*1* in this work. We first showed that IS*1*E transposed more frequently than IS*1*A (Figure 6B) although these elements carry the same consensus sequence (Figure 2A). Using a transcriptional *lacZ* reporter, we showed the upstream genomic region increased the levels of IS*1* transcription for both IS*1*Efs and IS*1*Afs, with more transcripts observed for IS*1*Efs than for IS*1*Afs (Figure 6D). These observations were confirmed by introducing a strong *rrnB* terminator (*rrnB*T) to block the upstream transcription (Figure 7B,D). Considering the similar effects found for both IS*5* and IS*1*, elevating transposase levels by transcribing through the downstream elements seems to be a common mechanism by which upstream host DNA segments affect transposition. A similar effect was observed for IS*1* inter-plasmid transposition, in which the transcripts of an upstream antibiotic-resistance gene were read through into the IS*1* sequence [56]. These observations are not surprising since most IS elements carry either weak or incomplete promoters driving their transposase genes [46,54,55,57].

Comparing the upstream DNA regions, there are two promoters (yhhZp5 and yhhZp7) that drive the *yhhZ*/*yhhA* operon and possibly the *insAB*’ gene of IS*1*E, as these genes are oriented in the same direction, and no terminator is present in the entire region (Figure 4A). However, there is a strong terminator present upstream of IS*1*A, which conceivably blocks the *rpsT* promoter from transcribing through the downstream IS*1* element (Figure 6A). The presence of different genomic contexts likely account for the observed discrepancy in transcription and transposition between two identical IS*1* elements (IS*1*E and IS*1*A). It would be interesting to determine if deleting the *rpsT* terminator has a positive effect on IS*1*A transposition. An increase in transposition would further indicate that upstream transcription levels can influence IS*1* transposition.

Disruption of the downstream region by insertion of a *km^r^* gene decreased IS*1*A transposition by over 3-fold (Figure 7F), indicating that the downstream genomic DNA is another genomic factor affecting IS*1* transposition. This effect should have nothing to do with the increased IS*1* transcripts. Instead, this downstream region adjacent to IS*1* may facilitate formation of the transpososome (critical for transposition), a nucleoprotein complex consisting of IS*1* and its transposase (and possibly some host factors such as IHF) [54,58,59]. Little information is available for formation of IS1 transpososomes. Disruption of the downstream region will likely limit the transposase (and the host factors) to access the region, thus disfavoring transpososome formation. These observed genomic impacts on IS*1* transposition are particularly noteworthy given the lack of prior experimental work involved in exploring any possible relationships between transposition of IS elements and their surrounding genomic DNA factors. To further test this model, different and additional edits could be made to the genomic regions surrounding both IS*1*A and IS*1*E. For example, to see if the downstream region has similar effects on other IS*1* elements, the same *km^r^* gene can be inserted downstream of IS*1*E. Other alterations, like adding a strong promoter, might also provide insight into the extent to which the genomic context may have on transposition rates.

IS*1* transposases are highly efficient in transposing IS*1* elements. Therefore, a stringent regulation of transposase production is critical to the host as frequent or deregulated transpositions are harmful due to inactivation of essential genes and abnormal alteration of genome stability, most likely leading to cell death. Stress-induced mutations are usually beneficial to bacterial cells, aiding them to survive harsh environments. During the past decade, increasing evidence has been provided to demonstrate that IS*1*-mediated mutations usually occur in response to specific environmental conditions such as starvation, and its transposition frequencies are under the control of host DNA-binding proteins and DNA structures [15,60,61,62]. An example of such stress-induced mutation takes place at the usually silent *bglGFB* operon, where IS*1* insertion leads to a positive β-glucoside growth (Bgl^+^) phenotype and preferentially occurs in the presence of a β-glucoside such as arbutin or salicin, and no other carbon source is available [63]. Another example involves IS*1* insertion into the *flhDC* regulatory region, which occurs preferentially within soft agar but not on hard agar or in liquid media and allows for the “swarming” phenotype [64,65,66]. Lastly, the *nfsA* and *nfsB* genes code for oxygen-insensitive nitroreductases, converting furazolidone into a toxic compound active against bacteria. When *nfsA* is lacking, IS*1* can transpose into *nfsB* in the presence of furazolidone, leading to a furazolidone resistance phenotype (FZD^+^) [7]. These observations support the notion that environment-directed, protein-regulated IS1 transpositions provide an advantage to host cells, enabling them to adapt to adverse conditions.

It is known that IS*1* transposition, either inter- or intramolecularly, can readily cause bacterial resistance to commonly used antibiotics by disrupting the native genes that encode proteins necessary to detoxify toxic compounds [7], or activating endogenous drug resistance genes by creating fusion promoters [13,14]. By flanking the *cat* gene (encoding a chloramphenicol acetyltransferase), two complete and identical IS*1* elements can noticeably mobilize *cat* from a Tn9 plasmid into the chromosome, conferring bacterial resistance to chloramphenicol [67]. Our ongoing preliminary studies reveal that a miniIS*1*, composed of a *cat* gene flanking by IS*1*’s inverted repeats (IRL and IRR), is capable of transposition within the *E. coli* genome. Together, these findings suggest a crucial role of IS*1* elements in both the emergence of multidrug resistance and the spread of antibiotic resistance genes among bacterial populations, especially in clinical isolates. Expression of the transposase gene (*insAB*’) is strictly regulated both transcriptionally (mediated by InsA) and translationally (mediated by ribosomal frameshift). Conceivably, any cellular or non-cellular conditions able to compromise such as stringent control would elevate IS*1*’s capability in developing and spreading drug resistance in bacterial cells. Part of these conditions include (i) a small cellular compound or metabolite produced under a specific condition may release InsA from the IS*1* promoter; (ii) a stress condition, such as the presence of UV, high temperature or starvation, may yield frequent point mutations on IS*1* elements, leading to the constitutive ribosomal frameshift and/or less InsA production; and (iii) some DNA-binding proteins or small RNAs, when available, may stabilize the pseudoknot structure near the poly-A tract, facilitating the ribosomes to perform the frameshift at the A_6_C motif. A better understanding of the mechanistic details for IS*1* transposition would help to develop new strategies to combat IS*1*-mediated multidrug resistance in clinical bacterial isolates.

In summary, we have demonstrated that endogenous IS*1* nucleotide sequences, intrinsic transcriptional and translational regulators, and the surrounding genomic contexts all significantly influence IS*1*’s transposition activity. Our findings highlight the complex relationship between sequence integrity, regulatory mechanisms, and genomic environment in modulating IS*1* mobility. Notably, these findings reveal a previously uncharacterized sensitivity of IS*1* transposition to genomic context. As transposable elements play critical roles in genome evolution and plasticity [1,68], understanding the factors that influence their activities remains a key priority. Future work will be necessary to further elucidate the molecular basis of these effects described above and assess their relevance across diverse genomic backgrounds. The findings revealed from the current work and further future work in this field will help refine our understanding of the tight and elegant regulation of IS elements activities.

## 4. Materials and Methods

### 4.1. E. coli Strains and Growth Conditions

Strain ZZ255, derived from *E. coli* K12 strain BW25113 [69], was used as the wildtype strain, in which four major copies of IS*5* (at *nmpC*, *wbbL*, *yejO*, and *gltI/lnt*) and the *lacI/lacZ/lacY* genes were deleted [33]. All other strains used in this study were derived from this strain, and they are described in (Table S1).

Bacterial strains were routinely cultured in LB media at 30 °C or 37 °C. For the β-glucoside growth (Bgl^+^) mutation assay, minimal medium M9 with 0.5% (*w*/*v*) β-glucoside as the sole carbon source was used. For furazolidone growth (FZD^+^) mutation assay and swarming (SWM^+^) mutation assay, LB media with appropriate alterations was used. For β-glucosidase assays, M9 with 0.5% glycerol was used. The 10 × M9 salt solution (per liter) contained 60 g of Na_2_HPO_4_, 30 g of KH_2_PO_4_, 10 g of NH_4_Cl, and 5 g of NaCl. After diluting to 1× M9 medium, it was supplemented with 1 mM MgSO_4_ and 0.1 mM CaCl_2_. When necessary, ampicillin (Ap), kanamycin (Km), and chloramphenicol (Cm) were added to the media at 100 μg/mL, 25 μg/mL, and 10 μg/mL, respectively.

### 4.2. Deletions of IS1 Elements from Chromosome

There are six IS*1* elements, classified as four different types based on their nucleotide sequences, across the chromosome of strain BW25113 at loci *yrhA*, *nhaR*, *yjhU*, *argF*, *afuB*, and *gfcA*, respectively. Using the Lambda Red approach [69], these elements were individually deleted by replacing the element with a kanamycin resistance gene (*km^r^*). Briefly, the *km^r^* gene flanked by FRT sequences was PCR amplified from pKD13 plasmid using a pair of chimeric oligos (Table S2), purified via gel electrophoresis and then electroporated into ZZ255 cells expressing Lambda Red proteins to replace the IS*1* element of interest via homologous recombination. Successful substitutions of *km^r^* for the IS element led to Km resistant phenotypes. Several Km-resistant colonies were subject to PCR verification. To remove the FRT-flanked *km^r^* gene, pCP20, coding for FLP recombinase, was introduced via electroporation, leaving behind an 85 bp FRT scar. These steps yielded six single IS*1* deletion mutants.

To combine multiple IS*1* deletions into one strain, we first used P1 transduction followed by flipping out the kmr gene and this two-step cycle was repeated until the desired deletion strains were achieved. Individual P1 phages were prepared using six single IS*1* deletions strains as the donor strains, each with an FRT-flanked *km^r^* gene in place of the target IS*1*. The standard P1 transduction approach was conducted to move an IS*1* deletion of interest into the Km-sensitive recipient strain [70].

To make IS1_A_, every other copy of IS*1* besides IS*1*A was removed with the above “P1 transduction/the *km^r^* gene “flipping-out” strategy such that only the copy of IS*1* at *nhaR* remained. To make IS1_E_, every other of IS*1* copy besides IS*1*E was removed such that only the copy of IS*1* at *yrhA* remained. To make IS1_AE_, four elements (IS*1*B, IS*1*C, IS*1*D and IS*1*F) (all except IS*1*A and IS*1*E) were deleted. To make ∆IS1, all IS*1* elements were deleted. Finally, to make ∆IS1_cons_, IS*1*A and IS*1*E were deleted (Table S1).

### 4.3. Construction of IS1fs at yrhA Locus and IS1fs at the nhaR Locus

To synthesize the transposases necessary for transposition, the ribosome must perform -1 translational frameshift at the A_6_C motif present in the middle of IS*1* transcripts. Without such frameshift, IS*1* only encodes InsA, the repressor for *insA* and *insAB’*. To see the effect of non-frameshift translation on IS*1* transposition, the 1st “A” at the A_6_C motif was altered to “G”, and another “G” was inserted between the 3rd and 4th “A” in the A_6_C motif in IS*1*E located at the *yrhA* locus.

To make such A_6_C to GA_2_GA_3_C alterations in strain IS1_E_ (retaining IS*1*E alone), a 400 bp genomic region, located at −463 To +95 relative to the 3’ end of IS*1*E, was first replaced by a *km^r^* gene amplified from the plasmid pKD13 [69] using primers IS1-fs-P1 and yrhD-fs-P2 (Table S2). The *km^r^* gene was flipped out by pCP20 leaving an 85 bp FRT scar to create the intermediate strain IS1_E_.fs-int1.

A 471 bp 3′ region of insAB’, harboring the modified A_6_C motif “GAAGAAAC” at the 5′ end, was PCR amplified using primers IS1-gg-F and yrhA-gg-R. Meanwhile, the *km^r^* gene was amplified from pKD13 using primers yrh-km-F and yrhD-fs-P2. These two fragments were fused together by PCR using primers IS1-int-F and yrhD-fs-P2. The resultant combined fragment was electroporated into the cells of IS1_E_.fs-int1 to replace the 85 bp FRT scar, yielding IS1_E_.fs-km, in which the modified IS*1* element, named IS1_E_.fs, no longer encodes the repressor InsA, and instead directly encodes the InsAB’ transposase. The gene was flipped out from IS1_E_.fs-km, yielding strain IS1_E_.fs.

Using a similar approach as described above, strains IS1_A_.fs-km and IS1_A_.fs were created with strain IS1_A_ (retaining IS*1*A only), in which IS1_A_.fs at the *nhaR* locus directly encodes InsAB’ but not InsA, the repressor for *insA*/*insAB’* transcription (Table S1).

### 4.4. Construction of P_tet_ Driving insA at the intS Locus

To construct P*_tet_* driving the repressor gene *insA*, the DNA region, located between +56 and +331 with regard to the 5′ end of IS*1*, was first amplified from IS1_AE_ genomic DNA using primers Ptet-AB-F and insA-int-P2 (Table S2). The amplified *insA* was linked to the 3′ end of the “*km^r^*:*rrnB*T:P*_tet_*” cassette by fusion PCR using primers intS-km-P1 and insAB-int-P2. The fusion product *km^r^*:*rrnB*T:P*_tet_*-*insAB* was chromosomally integrated to replace *intS*, yielding WT–InsA, in which P*_tet_* drives the transcription of *insA* at the *intS* locus. This construct was transferred to IS1_E_, IS1_E_.fs, and IS1_AE_.fs to create IS1_E_-InsA, IS1_E_.fs-InsA and IS1_AE_.fs -InsA.

To titrate *insA* expression, we transferred the *tetR* expression module (a strong constitutive promoter driving *tetR* at the *attB* site) [17] to strain IS1_E_.fs-InsA, yielding strain IS1_AE_.fs-AR. Titrated expression of *insA* is subject to the amount of the inducer, clTc (chlor-tetracycline), added to the media. Increasing amounts of clTc led to higher levels of *insA* expression, thus less frequent IS*1* transposition.

### 4.5. Construction of Transcriptional Reporters Using lacZ

To quantitate IS*1* transcription, a *lacZ* transcriptional reporter was constructed. The “*lacZ*:*cat*” cassette (composed of a promoter-less *lacZ* structural gene and a chloramphenicol-resistant gene cat with its promoter) plus its upstream *lacZ* ribosome binding site (RBS) was amplified from the genomic DNA of strain ZZ204 [71] using primers IS1-Z-P1 and yrhD-cat-P2 (Table S2). Using the Lambda-Red system, the amplified DNA fragments (that is, the “*lac*Z:*cat*” cassette plus *lacZ*’s RBS) was integrated immediately downstream of the stop codon TAA of the *insAB’* gene in strains IS1_A_.fs and IS1_E_.fs to replace the 15 bp IRR region of IS*1* and a short genomic region following the IS*1* element. This yielded two transcriptional operon reporter strains: IS1_A_.fs and IS1_E_.fs, in which *insAB’* and *lacZ* formed an operon and its transcription was driven by P_IS1_. In each of these reporter, two separate proteins, InsAB’ and LacZ, were made, and the latter can be quantitated by β-galactosidase assays.

### 4.6. Addition of rrnB Terminator

Using the aforementioned Lambda Red approach, a strong terminator, the *rrnB* terminator, was added directly upstream of IS*1*A in strains IS1_A_ and IS1_A_.fs. The selection markers were subsequently flipped out by pCP20, creating strains IS1_A_.fs-T and IS1_A_-T, respectively (Table S1). Similarly, strains IS1_E_-T and IS1_E_.fs-T were made by inserting the *rrnB* terminator upstream of IS*1* in strains IS1_E_ and IS1_E_.fs, respectively. The presence of the terminator would prevent upstream transcription from contributing to IS*1* transposase transcription.

### 4.7. β-Glucoside Growth Mutation (Bgl^+^) Assay

This mutation assay was the primary method for quantifying IS*1* transposition frequency/activity for our experimental strains, performed as previously reported by our group and others [17,33,60]. The assay was conducted on minimal M9 agar plates with 0.5% salicin (a β-glucoside) as the only carbon source. Prior to plating-day (Post-plate Day 0), these M9/Salicin plates were prepared by autoclaving a 1.68% water-agar solution, after which the appropriate amount of 10× M9 salts and 5% Salicin were added to create a final solution of 1.5% agar and 0.5% salicin. Plates were poured the same day, 25 mL of agar solution per plate. Early on plating-day, a fresh colony from each experimental strain was cultured in LB liquid media in a 37 °C water shaking bath for about 8 h. After the OD_600_ was measured, the cells were washed twice using 1× M9 salt (carbon source-free) and diluted to the plating concentration and applied onto agar plates (2 × 10^7^ or 2 × 10^6^ cells per plate depending on the strain). At least four plates were used for each strain in each experiment. The same experiment was repeated at least twice. The plates were then incubated in a 30 °C incubator and were examined daily for the appearance of Bgl^+^ colonies, with each colony representing a new Bgl^+^ mutation. Any colonies appearing prior to day 2 post-plating were considered to be Bgl^+^ cells initially applied onto the plates and thus subtracted from the subsequent measurements. The background populations (total number of Bgl^−^ cells) were determined as previously [72,73,74]. The frequency of Bgl^+^ mutations were determined by dividing the total Bgl^+^ colonies by the number of Bgl^−^ cells plated and normalized to Bgl^+^ mutations per 10^8^ cells at any given time point.

### 4.8. Swarming Mutation (SWM^+^) Assay

This mutation assay was used as a secondary method to measure IS*1* transposition frequencies in our experimental strains, performed following a previously documented approach [66]. On plating-day, a fresh colony from each experimental strain was cultured in LB liquid media in a 37 °C water shaking bath for about 8 h. LB soft agar (0.3% *w*/*v*) plates were prepared and were used in the same plating-day. Once ready, the culture was washed once with 1× M9 salts and diluted to an OD_600_ of 1.0. Three separate injections of 1.5 µL of the cell suspension were inoculated into one LB semisolid agar plate, each containing 1.5 × 10^6^ cells. At least four plates were used for each assay replication. The plates were incubated at 30 °C for 22 h. The SWM^+^ mutants were represented by outgrowths of motile subpopulations from the injection sites and counted.

### 4.9. Furazolidone Resistance Mutation (FZD^+^) Assay

This mutation assay required prior mutation of the *nfsA* gene, which was either acquired from previous studies [60] or made via P1 transduction.

This mutation assay was used as another secondary method to quantifying IS*1* transposition frequencies, performed following a previously recorded approach [66]. Prior to plating-day (Post-plate Day 0), LB + FZD (6.5 μg/mL) agar plates were prepared. Cell suspensions used for plating were prepared as for Bgl^+^ mutation assays and were applied onto agar plates at 10^8^ cells per plate. The plates were incubated in a 30 °C incubator and were examined daily for the appearance of FZD^+^ colonies, with each colony representing a new FZD^+^ mutation. The frequencies of FZD*^+^* mutations were determined by dividing the total FZD^+^ colonies by the number of FZD^−^ cells plated and normalized to FZD^+^ mutations per 10^8^ cells at any given time point.

### 4.10. β-Galactosidase Assay

β-galactosidase assays were conducted as previously reported [54]. Briefly, test strains were cultured in M9 glycerol minimal media. During the exponential growth phase, fives samples per strain were collected in an OD_600_ range of 0.2 to 1.0. The experiment was repeated at least twice. β-galactosidase activity for each sample was measured using o-nitrophenyl-β-D-galactopyranoside (β-ONPG) as the substrate. The slope of β-galactosidase activities versus the collected OD_600_ values for each sample represents the reporter strain activity.

### 4.11. Statistical Analysis

All IS*1* mutation frequency data are expressed as the mean ± standard deviation (SD). Mutation frequencies were calculated in Google Sheets. Statistical significance was tested by either Welch’s or unpaired *t*-test (for 2 treatments) or a Welch’s or regular one-way ANOVA followed by Tukey multiple comparisons test, Games-Howell multiple comparisons test or Dunnett T3 multiple comparisons test, or a Kruskal–Wallis with Dunn’s multiple comparisons test (for ≥3 treatments) using BioRender. Details of the statistical tests used are indicated in each figure legend. All figures and figure panels were created in BioRender; https://BioRender.com. NS denotes no significance and indicates a *p*-value ≥ 0.05; * indicates a *p*-value < 0.05; ** indicates a *p*-value < 0.01; *** indicates a *p*-value < 0.001; and **** indicates a *p*-value < 0.0001.

## Data Availability

The data presented in this study are available upon request from the corresponding authors.

## Supplementary Materials

The following supporting information can be downloaded at: https://www.mdpi.com/article/10.3390/ijms26178375/s1.
