# Supplementary material for: Examining the Roles of Genomic Context and Endogenous Regulatory Elements on IS1 Transposition Within the Escherichia coli Genome
Source: Int J Mol Sci. 2025 Aug 28;26(17):8375. doi: 10.3390/ijms26178375 (PMC12428005; doi:10.3390/ijms26178375)
Supplement: Supplementary file 1 [file ijms-26-08375-s001.zip › ijms-3820652-supplementary.pdf]

IRL (30bp)

O_{InsA} or $O_{InsAB'}$

ggtgatgctgccaacttactgatttagtgtatgatggtgtttttgaggtgctcca**GTG**gcttctgtttctatcagctgtccctcct

-35 O_{IHF} -10 Start codon for *insA* or *insAB'*

gttcagctactgacggggtggtgcgtaacggcaaaagcaccgccggacatcagcgctatctctgctctcactgccgtaaaacatgg
caactgcagttcacttacaccgcttctcaacccggtacgcaccagaaaatcattgatatggccatgaatggcgttgg**atg**ccgggc

Start codon for *insB*

aacagcccgcattatgggcgttggcctcaacacgattttacgtcacttaaaaaactcaggccgcagtcggtaacctcgcgcataca

A_6C Stop codon for *insA*

gccgggcagtgacgtcatcgtctgcgcggaaatggacgaacagtggggctatgtcggggctaaatcgcgccagcgctggctgtttt
acgcgtatgacagtctccggaagacggttgttgcgcacgtattcggtgaacgcactatggcgacgctggggcgtcttatgagcctg
ctgtcaccctttgacgtggtgatatggatgacggatggctggccgctgtatgaatcccgcctgaagggaaagctgcacgtaatcag
caagcgatatacgcagcgaattgagcggcataacctgaatctgaggcagcacctggcacggctgggacggaagtcgctgtcgttct

O_{InsA} or $O_{InsAB'}$

O_{IHF}

caaaatcggtggagctgcatgacaaagtcatcgggcattatctgaacataaa**acactatcaaTAAgttggagtcattacc**

Stop codon for *insB* or *insAB'*

IRR (28 bp)

Figure S1. IS1A/IS1E nucleotide sequence with annotations. IRL and IRR flanking the element are bolded. The -35 hexamer and -10 hexamer for P_{IS1} are green highlighted. The InsA or InsAB' binding sites overlapping with IRL and IRR are overlined. The IHF binding site is under dot-lined at IRL or is over dot-lined at IRR. The start codons and stop codons for InsA and InsAB' are individually labelled. The A_6C motif for ribosomal frameshift is cyan highlighted.

```
             *         *         *         *         *          *         *
  1>GGTGATGCTGCCAACTTACTGATTTAGTGTATGATGGTGTTTTTGAGGTGC-TCCAgtgGCTTCTGTTTCTATCAGCTGT>79
  1>GGTGATGCTGCCAACTTACTGATTTAGTGTATGATGGTGTTTTTGAGGTGC-TCCAgtgGCTTCTGTTTCTATCAGCTGT>79
  1>GGTGATGCTGCCAACTTACTGATTTAGTGTATGATGGTGTTTTTGAGGTTC-TCCAgtgGCTTCTGTTTCTATCAGCTGT>79
  1>GGTAATGCTGCCAACTTACTGATTTAGTGTATGATGGTGATTTTAAGGTGCTTGC-gtgGCTTCCATTTCCATCAGATGT>79

    *         *         *         *         *         *         *         *
 80>CCCTCCTGTTCAGCTACTGACGGGGTGGTGCGTAACGGCAAAAGCACCGCCGGACATCAGCGCTATCTCTGCTCTCAC-T>158
 80>CCCTCCTGTTCAGCTACTGACGGGGTGGTGCGTAACGGCAAAAGCACTGCCGGACATCAGCGCTATCTCTGCTCTCAC-T>158
 80>CCCTCCTGTTCAGCTACTGACGGGGTGGTGCGTAACGGCAAAAGCACTGCCGGACATCAGCGCTATCTCTGCTCTCAC-T>158
 80>CCTTCCTGCTCCGCTACTGAAGGCGTGGTGCGTAACGGCAAAAGCACTGCCGGACATCAGCGCTATCTCTGCTCTC-CTT>158

     *         *         *         *         *         *         *         *
159>GCCGTAAAACATGGCAACTGCAGTTCACTTACACCGCTTCTCAACCCGGTACGCACCAGAAAATCATTGATATGGCCATG>238
159>GCCGTAAAACATGGCAACTGCAGTTCACTTACACCGCTTCTCAACCCGGTACGCACCAGAAAATCATTGATATGGCCATG>238
159>GCCGTAAAACATGGCAACTGCAGTTCACTTACACCGCTTCTCAACCCGGTACGCACCAGAAAATCATTGATATGGCCATG>238
159>GCCGTAAAACATGGCAACTACAGTTCACTTACACCGCCTCTCAGCCCGGTAAGCACCAGAAAATCATTGATATGGCCATG>238

     *         *         *           *         *         *         *         *
239>AATGGCGTTGGATGCCGGGCAACAG-CC-CGCATTATGGGCGTTGGCCTCAACACGATTTTACGTCACTTAAAAAACTCA>316
239>AATGGCGTTGGATGCCGGGCAACCG-CC-CGCATTATGGGCGTTGGCCTCAACACGATTTTCCGCCATTTAAAAAACTCA>316
239>AATGGCGTTGGATGCCGGGCAACAG-CC-CGCATTATGGGCGTTGGCCTCAACACGATTTTCCGCCATTTAAAAAACTCA>316
239>AATGGCGTCGGATGTCGCGC--CAGTGCACGCATTATGGGCGTTGGCCTCAACACGGTTTACGTCACTTAAAAAACTCA>316

       *         *         *         *         *         *         *         *
317>GGCCGCAGTCGGTAACCTCGCGCATACAGCCGGGCAGTGACGTCATCGTCTGCGCGGAAATGGACGAACAGTGGGGCTAT>396
317>GGCCGCAGTCGGTAACCTCGCGCATACAGCCGGGCAGTGACGTCATCGTCTGCGCGGAAATGGACGAACAGTGGGGATAC>396
317>GGCCGCAGTCGGTAACCTCGCGCATACAGCCGGGCAGTGACGTCATCGTCTGCGCGGAAATGGACGAACAGTGGGGATAC>396
317>GGCCGCAGTCGGtaaCCTCGCGCATACAACCGGGCAGTGATGTGATTGTCTGCGCTGAAATGGACGAACAGTGGGGCTAC>396

       *         *         *         *         *         *         *         *
397>GTCGGGGCTAAATCGCGCCAGCGCTGGCTGTTTTACGCGTATGACAGTCTCCGGAAGACGGTTGTTGCGCACGTATTCGG>476
397>GTCGGGGCTAAATCGCGCCAGCGCTGGCTGTTTTACGCGTATGACAGGCTCCGGAAGACGGTTGTTGCGCACGTATTCGG>476
397>GTCGGGGCTAAATCGCGCCAGCGCTGGCTGTTTTACGCGTATGACAGGCTCCGGAAGACGGTTGTTGCGCACGTATTCGG>476
397>GTCGGTGCTAAATCACGTCAGCGCTGGCTGTTTTACGCGTATGACAGGATACGGAGGACGGTTGTGGCGCACGTCTTCGG>476

       *         *         *         *         *         *            *         *
477>TGAACGCACTATGGCGACGCTGGGGCGTCTTATGAGCCTGCTGTCACCCTTTGA---CGTGGTGATATGGATGACGGATG>553
477>TGAACGCACTATGGCGACGCTGGGGCGTCTTATGAGCCTGCTGTCACCCTTTGA---CGTGGTGATATGGATGACGGATG>553
477>TGAACGCACTATGGCGACGCTGGGGCGTCTTATGAGCCTGCTGTCACCCTTTGA---CGTGGTGATATGGATGACGGATG>553
477>TGAACGCACTCTGGCCACACTGGAGCGTCTTCTGAGCCTGCTGTCGGCCTTTGAGGTCGTGGT-ATA--GATGACGGATG>553

          *         *         *         *         *         *         *         *
554>GCTGGCCGCTGTATGAATCCCGCCTGAAGGGAAAGCTGCACGTAATCAGCAAGCGATATACGCAGCGAATTGAGCGGCAT>633
554>GCTGGCCGCTGTATGAATCCCGCCTGAAGGGAAAGCTGCACGTAATCAGCAAGCGATATACGCAGCGAATTGAGCGGCAT>633
554>GCTGGCCGCTGTATGAATCCCGCCTGAAGGGAAAGCTGCACGTAATCAGCAAGCGATATACGCAGCGAATTGAGCGGTAT>633
554>GCTGGCCGCTGTATGAATCACGCCTGAAGGGAAAGCTGCACGTTATCAGCAAGCGTTACACTCAGCGCATTGAGCGACAT>633

          *         *         *         *         *         *         *         *
634>AACCTGAATCTGAGGCAGCACCTGGCACGGCTGGGACGGAAGTCGCTGTCGTTCTCAAAATCGGTGGAGCTGCATGACAA>713
634>AACCTGAATCTGAGGCAGCACCTGGCACGGCTGGGACGGAAGTCGCTGTCGTTCTCAAAATCGGTGGAGCAGCATGACAA>713
634>AACCTGAATCTGAGGCAGCACCTGGCACGGCTGGGACGGAAGTCGCTGTCGTTCTCAAAATCGGTGGAGCTGCATGACAA>713
634>AATCTGAATCTGAGACAACATCTGGCAAGGCTGGGACGGAAGTTACTGTCGTTCTCAAAATCGGTGGAGTTGCATGACAA>713

          *         *         *         *         *
714>AGTCATCGGGCATTATCTGAACATAAAACACTATCAAtaaGTTGGAGTCATTACC>768
714>AGTCATCGGGCATTATCTGAACATAAAACACTATCAAtaaGTTGGAGTCATTACC>768
714>AGTCATCGGGCATTATCTGAACATAAAACACTATCAAtaaGTTGGAGTCATTACC>768
714>GGTCATCGGGCATTATCTGAACATAAAACACTATCAGTAAGTTGGAGTCATTACC>768
```

Figure S2. Nucleotide sequence alignment of the four IS*1* element variants. For each panel, the sequences from the 1st line to the 4th line refer to the sequences from IS*1*A/IS*1*E, IS*1*B/IS*1*C, IS*1*D and IS*1*F, respectively. Mismatches, deletions or insertions are red highlighted. Created in BioRender. Smith, S. (2025) https://BioRender.com/9oq5ig8.

Table S1. Strains used in this study

**Strain Name**	**Description**	**Source**
BW25113	Primary background strain	[1]
WT	BW25113 deleted for four primary IS*5* copies and *lacI*/*lacZ*/*lacY*	[2]
ΔIS1$_{cons}$	ΔIS*1*A and ΔIS*1*E	This Study
IS1$_{A}$	Retaining IS*1*A only (ΔIS*1*B, ΔIS*1*C, ΔIS*1*D, ΔIS*1*E and ΔIS*1*F)	This Study
IS1$_{E}$	Retaining IS*1*E only (ΔIS*1*A, ΔIS*1*B, ΔIS*1*C, ΔIS*1*D and ΔIS*1*F)	This Study
IS1$_{AE}$	Retaining IS*1*A and IS*1*E only (ΔIS*1*B, ΔIS*1*C, ΔIS*1*D and ΔIS*1*F)	This Study
ΔIS1	IS*1* free (ΔIS*1*A, ΔIS*1*B, ΔIS*1*C, ΔIS*1*D, ΔIS*1*E and ΔIS*1*F)	This Study
IS1$_{E}$.fs	GAAGAAC replacing the A_6C motif at IS*1*E in strain IS1$_{E}$	This Study
IS1$_{AE}$.fs	GAAGAAC replacing the A_6C motif at IS*1*E in strain IS1$_{AE}$	This Study
IS1$_{E}$.fs-InsA	IS1$_{E}$.fs with *insA* overexpression (P_{tet} driving *insA* at the *intS* locus)	This Study
IS1$_{AE}$.fs -InsA	IS1$_{AE}$.fs with *insA* overexpression (P_{tet} driving *insA* at the *intS* locus)	This Study
IS1$_{E}$-InsA	IS1$_{E}$ with *insA* overexpression (P_{tet} driving *insA* at the *intS* locus)	This Study
IS1$_{E}$.fs-AR	IS1$_{E}$.fs with P_{tet}-driven *insA* and constitutive expression of *tetR*	This Study
IS1$_{A}$.fs	GAAGAAC replacing the A_6C motif at IS*1*E in strain IS1$_{A}$	This Study
IS1$_{A}$.fs–Z	Transcriptional reporter with a *lacZ* gene (including its RBS) integrated downstream of the *insAB'* gene in IS1$_{A}$.fs	This Study
IS1$_{E}$.fs–Z	Transcriptional reporter with a *lacZ* gene (including its RBS) integrated downstream of the *insAB'* gene in IS1$_{E}$.fs	This Study
IS1$_{A}$.fs-km	km^r insertion downstream of IS*1*Afs in strain IS1$_{A}$.fs	This Study
IS1$_{A}$-T	Strain IS1$_{A}$ with a *rrnB* terminator inserted just upstream of IS*1*A	This Study
IS1$_{E}$ -T	Strain IS1$_{E}$ with a *rrnB* terminator inserted just upstream of IS*1*E	This Study
IS1$_{A}$.fs -T	Strain IS1$_{A}$.fs with a *rrnB* terminator inserted just upstream of IS*1*Afs	This Study
IS1$_{E}$.fs-T	Strain IS1$_{E}$.fs with a *rrnB* terminator inserted just upstream of IS*1*Efs	This Study

Table S2. Oligonucleotides used in this study

Name	Sequence	Use
Pbgl-F2	tggcgatgagctggataaactgctg	Verification for IS*1* insertion into *bglGFB*
Pbgl-R2	tcagttcatgactgctcaaggcatac	Verification for IS*1* insertion into *bglGFB*
nfsB-ver-Fn	aagaaatctccgaagcgttacttcg	Verification for IS*1* insertion into *nfsB*
nfsB-ver-Rn	agggttatgcaaatcaggagaatctg	Verification for IS*1* insertion into *nfsB*
PflhDC-Xho-F	atactcgagcttattctgtgaacttcaggtgac	Verification for IS*1* insertion into *flhDC*
PflhDC-Bam-R	aatggatcccatagcggacgctttgtcctgaac	Verification for IS*1* insertion into *flhDC*
IS1F-P1	ggtgatgctgccaacttactgatttagtgtatgatggtgtttttgaggtgctgtgtaggctggagctgcttc	Replacing IS*1* with km^r to make individual IS1 deletion mutants
nhaR-IS1-P2	cttgagacattgtttccatatgtacgcgggcgaataaatagaggaatctgattccggggatccgtcgacctg	Replacing IS*1*A with km^r
nhaR-ver-R	agatagattagttgtacattaccacg	Verification of km^r substitution for IS*1*A
afu-IS1-P2	cgcagggaaccggcgcgcaggctgagtgaggcttcatcgagcgatttatcgattccggggatccgtcgacctg	Replacing IS*1*B with km^r
afuB-ver-F	agctgtaaatcagcgccgagaggatc	Verification of km^r substitution for IS*1*B
argF-IS1-P2	attatgcttctaaaatggcggagaacatggcgaggcttgctgccttacttcattccggggatccgtcgacctg	Replacing IS*1*C with km^r
afgF-ver-F	aggattatgcttctaaaatggcggag	Verification of km^r substitution for IS*1*C
csdH-IS1-P2	ttcacaaggatgttatactcaaagtagacaatcctgatctcaagcgtacgtattccggggatccgtcgacctg	Replacing IS*1*D with km^r
csdH-ver-R	tatgctcacaagtcgtatttccagag	Verification of km^r substitution for IS*1*D
yrhD-IS1-P2	gagaaatcactaaacgaactgaatatattttctgtgccaatattatctctaattccggggatccgtcgacctg	Replacing IS*1*E with km^r
yrhD-ver-R	tattatgaacaactgtccatgatttcg	Verification of km^r substitution for IS*1*E
yihU-IS1-P2	tatggggcaggattttattgattagcggcgataataagaacaatacccacaattccggggatccgtcgacctg	Replacing IS*1*F with km^r
yihU-ver-R	tgattgttgatgaaggaacggcagag	Verification of km^r substitution for IS*1*F
IS1-fs-P1	caacagcccgcattatgggcgttggcctcaacacgattttacgtcacttgtgtgtaggctggagctgcttc	Construction of frameshift strain $IS1_E$.fs
yrhD-fs-P2	ggtttacaatgatttttgtttcctttttgattattatgaacaactgtccatgattccggggatccgtcgacctg	Construction of frameshift strain $IS1_E$.fs
IS1-gg-F	ctcaacacgattttacgtcacttgaagaaactcaggc	Construction of frameshift strain $IS1_E$.fs
yrhA-gg-R	gaagcagctccagcctacacatttcgtttaagaatgaagagaaatcac	Construction of frameshift strain $IS1_E$.fs
yrh-km-F	gtgatttctcttcattcttaaacgaaatgtgtaggctggagctgcttc	Construction of frameshift strain $IS1_E$.fs
intS-P1	agatttacagttcgtcatggttcgcttcagatcgttgacagccgcactccatgtgtaggctggagctgcttc	Construction of P_{tet}-driven *insA* at *intS*
AB-Ptet-R	acagctgatagaaacagaagccaccatggtacctttctcctctttaatgaattc	Construction of P_{tet}-driven *insA* at *intS*
Ptet-AB-F	gaattcattaaagaggagaaaggtaccatggtggcttctgtttctatcagctgtcc	Construction of P_{tet}-driven *insA* at *intS*
insA-int-P2	gttgttaaggtcgctcactccaccttctcatcaagccagtccgcccaccattgcatcattaccgactgcggcctgagttctttaagtg	Construction of P_{tet}-driven *insA* at *intS*

IS1-fs-P1	caacagcccgcattatgggcgttggcctcaacacgattttacgtcacttgtgtgtaggctggagctgcttc	Construction of frameshift strain $IS1_A$.fs
nhR-fs-P2	aggtgggggagatagattagttgtacattaccacgattttgactcggctcattccggggatccgtcgacctg	Construction of frameshift strain $IS1_A$.fs
IS1-gg-F	ctcaacacgattttacgtcacttgaagaaactcaggc	Construction of frameshift strain $IS1_A$.fs
nhR-gg-R	gaagcagctccagcctacacatgcccgcttgagacattgtttccatatgtac	Construction of frameshift strain $IS1_A$.fs
nhR-km-F	gtacatatggaaacaatgtctcaagcgggcatgtgtaggctggagctgcttc	Construction of frameshift strain $IS1_A$.fs
IS1-int-F	gatgccgggcaacagcccgcattatgggcgttggcctcaacacgattttacgtcacttg	Construction of frameshift strain $IS1_A$.fs
IS1A-Z-P1	ctgcatgacaaagtcatcgggcattatctgaacataaaacactatcaataacaggaaacagctatgaccatgattacggattc	*lacZ* transcriptional reporter strain $IS1_A$.fs-Z
nhaR-cat-P2	aggtgggggagatagattagttgtacattaccacgattttgactcggctcattacgccccgccctgccactcatcgcagtac	*lacZ* transcriptional reporter strain $IS1_A$.fs-Z
IS1E-Z-P1	gagctgcatgacaaagtcatcgggcattatctgaacataaaacactatcaataacaggaaacagctatgaccatgattacggattc	*lacZ* transcriptional reporter strain $IS1_E$.fs-Z
yrhA-cat-P2	ggtttacaatgatttttgtttccttttgattattatgaacaactgtccatgattacgccccgccctgccactcatcgcagtac	*lacZ* transcriptional reporter strain $IS1_E$.fs-Z
rpsT-T-F	cctcccatgaaaaatattatggaccacacaaaatgatgactaaaacccaaatgtgtaggctggagctgcttc	*rrnB*T insertion upstream of IS*1*A or IS*1*Afs
nhaR-T-R	atcagccgatggttctacgattcttaagccacgaagagttcagatagtacaaaggttcatcgcgctcgagacgca	*rrnB*T insertion upstream of IS*1*A or IS*1*Afs
yrhA-T-F	cctcccatgaaaaatattatggaccacacaaaatgatgactaaaacccaaatgtgtaggctggagctgcttc	*rrnB*T insertion upstream of IS*1*E or IS*1*Efs
yrhA-T-R	ttatactttcaataaatgattccttgagcggtgcttcaaatggatagtctaaaggttcatcgcgctcgagacgca	*rrnB*T insertion upstream of IS*1*E or IS*1*Efs

References

1. Datsenko, K.A.; Wanner, B.L. One-Step Inactivation of Chromosomal Genes in Escherichia coli K-12 Using PCR Products. *Proc. Natl. Acad. Sci. USA* **2000**, *97*, 6640–6645.
2. Vögele, K.; Schwartz, E.; Welz, C.; Schiltz, E.; Rak, B. High-Level Ribosomal Frameshifting Directs the Synthesis of IS150 Gene Products. *Nucleic Acids Res.* **1991**, *19*, 4377–4385.
